# Trehalose metabolism genes render rice white tip nematode *Aphelenchoides besseyi* (Nematoda: Aphelenchoididae) resistant to an anaerobic environment

**DOI:** 10.1242/jeb.171413

**Published:** 2018-02-15

**Authors:** Qiaoli Chen, Feng Wang, Danlei Li, Ruizhi Zhang, Yaming Ling

**Affiliations:** College of Forestry, Northeast Forestry University, Harbin, Heilongjiang 150040, China

**Keywords:** Stress resistance, Anoxybiosis, Re-aeration, Trehalase, Trehalose-6-phosphate synthase

## Abstract

After experiencing anaerobic environments, *Aphelenchoides besseyi* will enter a state of suspended animation known as anoxybiosis, during which it may use trehalose as an energy supply to survive. To explore the function of trehalose metabolism, two trehalose-6-phosphate synthase (TPS) genes (*Ab-tps1* and *Ab-tps2*) encoding enzymes catalysing trehalose synthesis, and three trehalase (TRE) genes (*Ab-ntre1*, *Ab-ntre2* and *Ab-atre*) encoding enzymes catalysing the hydrolysis of trehalose, were identified and investigated. *Ab-tps1* and *Ab-tps2* were active during certain periods of anoxybiosis for *A. besseyi*, and *Ab-tps2*, *Ab-ntre1*, *Ab-ntre2* and *Ab-atre* were active during certain periods of recovery. The results of RNA interference experiments suggested that *TRE* genes regulated each other and both TPS genes, while a single TPS gene only regulated the other TPS gene. However, two TPS genes together could regulate TRE genes, which indicated a feedback mechanism between these genes. All these genes also positively regulated the survival and resumption of active metabolism of the nematode. Genes functioning at re-aeration have a greater impact on nematode survival, suggesting that these genes could play roles in anoxybiosis regulation, but may function within restricted time frames. Changes in trehalose levels matched changes in TRE activity during the anoxybiosis–re-aeration process, suggesting that trehalose may act as an energy supply source. The observation of up-regulation of TPS genes during anoxybiosis suggested a possible signal role of trehalose. Trehalose metabolism genes could also work together to control trehalose levels at a certain level when the nematode is under anaerobic conditions.

## Abstract (precis)

**Summary:** To ensure survival, nematodes utilize both extracellular and intracellular trehalose, and trehalose metabolism genes regulate each other to keep trehalose and trehalase activity at certain levels during the anoxybiosis–re-aeration process.

## INTRODUCTION

Rice white tip nematode (*Aphelenchoides besseyi* Christie 1942) is a parasite in more than 200 plants in 35 genera. Rice (*Oryza sativa*) and strawberry (*Fragaria*×*ananasa*) are its most common hosts (Franklin and Siddiqi, 1972; Bridge et al., 1990; Wang et al., 1993; Tsay et al., 1995). It causes serious diseases in rice, and decreases rice yield by 10–20% in general and over 30% in severe cases, which results in large annual economic losses worldwide (Duncan et al., 2006).

Nematodes possess no respiratory and circulatory systems, and their internal oxygen levels are mainly determined by ambient oxygen levels and body size (Qiu and Bedding, 2000). It has been reported that there is no detectable oxygen in the soil at a depth of 61 cm immediately after irrigation (Van Gundy et al., 1968). Presumably, soil porosity, water content, temperature, groundwater depth, microbial respiration and even every heavy rain will affect oxygen level changes in soil (Sierra and Renault, 1998). Most animals and plants die when they experience anaerobic conditions that cause low internal oxygen levels. However, *A. besseyi* can enter a state of suspended animation known as anoxybiosis after experiencing anaerobic environments, and this allows its survival. During anoxybiosis, the nematode is inactivated but can survive for several days in an immobile state; upon re-aeration the nematode will resume ‘normal’ metabolism (Cooper and Van Gundy, 1970). The ability of *A. besseyi* to survive under anaerobic conditions makes it difficult to prevent and control the infestations caused by these nematodes.

Several studies on the oxygen requirements of nematodes during development and storage have been reported (Qiu and Bedding, 2000). Anoxybiosis is often associated with trehalose (Qiu and Bedding, 2000). Trehalose is a natural non-specific cell protection material; it is claimed to be important in the physiology of nematodes where it may function in sugar transport, energy storage and protection against environmental stresses (Pellerone et al., 2003). As a stress protectant, trehalose is thought to act by preserving lipid membranes and stabilizing proteins in their native state (Singer and Lindquist, 1998; Crowe and Crowe, 2000; Guo et al., 2000). However, it has been reported that under anaerobic conditions, the nematode *Steinernema carpocapsae* was immobile and its glycogen and trehalose content decreased sharply while lipid and protein content did not change substantially (Qiu and Bedding, 2000). When anaerobically incubated *S. carpocapsae* was returned to an aerobic environment, both glycogen and trehalose levels increased while lipid levels decreased sharply (Qiu and Bedding, 2000). Like most other free-living nematodes, *S. carpocapsae* cannot use lipids and depends on anaerobic degradation of their carbohydrate reserves, mainly glycogen and trehalose, for energy supply (Tielens and Van den Bergh, 1993). Inspired by former studies regarding the crucial roles trehalose plays in protection and energy supply, functions of trehalose-related genes of the harmful plant parasitic nematode *A. besseyi* were investigated here to study its energy metabolism and the relationships of related genes to the ability of survival under anaerobic conditions.

In most eukaryotes, trehalose is catalysed by three trehalose metabolism enzymes. Trehalose-6-phosphate synthase (TPS) (Vandesteene et al., 2010; Li et al., 2011; Sengupta et al., 2011a,b; Delorge et al., 2015) and trehalose-6-phosphate phosphatase (TPP) (Ponnu et al., 2011; Vandesteene et al., 2012; Lahiri et al., 2014; Yadav et al., 2014) are responsible for trehalose synthesis (Avonce et al., 2010); trehalase (TRE) catalyses the hydrolysis of trehalose (Pellerone et al., 2003; Hespeels et al., 2015), and regulates the concentration of sugars (Alabran et al., 1983; Behm, 1997). It has been reported that trehalose hydrolysis is catalysed by two types of TRE, acid TRE (AT; Dmitryjuk and Zółtowska, 2003) and neutral TRE (NT; Londesborough and Varimo, 1984). AT and NT are responsible for utilization of extracellular trehalose and mobilization of intracellular trehalose, respectively (Londesborough and Varimo, 1984; Basu et al., 2006). By hydrolysing trehalose, the various tissues and organs will obtain glucose, effectively protecting cells in the body as their resilience is enhanced (Behm, 1997). As the only hydrolase that specifically hydrolyses trehalose into glucose, TRE is proposed to be the key enzyme of trehalose metabolism (Dmitryjuk et al., 2006). Based on research into the function of different types of TRE (Alabran et al., 1983; Sengupta et al., 2011a,b; Van Houtte et al., 2013), as well as the research and application of enzyme inhibitors, TRE has become a new potential target of plant parasitic nematode control. A correlation between regulation of trehalose synthesis and survivability of the organism under thermal stress has been established in *Candida utilis* (Lahiri et al., 2014). However, so far no TPP genes have been identified in *A. besseyi* or other nematodes (Pellerone et al., 2003; Hespeels et al., 2015). For these reasons, the study reported here was restricted to the transcript levels of TRE and TPS genes and the trehalose levels and activity of AT or NT to investigate the trehalose metabolism of *A. besseyi* under anaerobic conditions.

Trehalose metabolism genes, including TRE and TPS genes, have been cloned from numerous plants and microorganisms (Bansal et al., 2013; Ge et al., 2011; Hespeels et al., 2015; Müller et al., 2001; Pellerone et al., 2003). Two TPS genes (*Ab-tps1*, NCBI accession number KY661388; *Ab-tps2*, NCBI accession number KY661389) and a TRE gene (*Ab-tre*, NCBI accession number KY661390, which was further identified as a NT-encoding gene) were active at certain periods when *A. besseyi* were entering or recovering from osmobiosis (Qiaoli et al., 2017), which manifested the essential roles of trehalose metabolism genes in *A. besseyi* undergoing hypertonic osmotic pressure. A functional analysis of trehalose metabolism genes in *A. besseyi* under anaerobic environments will shed light on the mechanism of trehalose metabolism for anaerobic tolerance within nematodes and provide new ideas for rice white tip nematode biological control.

## MATERIALS AND METHODS

### Identification of homologous TPS- and TRE-encoding genes

The tool tblastn (https://blast.ncbi.nlm.nih.gov/Blast.cgi) was used to select homologous TPS- and TRE-encoding genes from the *A. besseyi* transcriptome (Wang et al., 2014) based on homologous TPS and TRE of *Caenorhabditis elegans* (Pellerone et al., 2003). To identify types of TREs (NT or AT), SignalP4 (http://www.cbs.dtu.dk/services/SignalP/) was used to identify signal peptides; Motif scan (http://myhits.isb-sib.ch/cgi-bin/motif_scan) was used to analyse the protein motifs; ScanProsite (http://prosite.expasy.org/) was used to analyse structure characteristics of proteins; and PSORT II Prediction (http://psort.hgc.jp/form2.html) was used to predict the subcellular localizations.

### Nematodes and gene cloning

The nematode *A. besseyi* (NCBI BioSample accession number SAMN02420038) was cultured on *Botrytis cinerea* at 25°C in the dark. Nematodes freshly extracted using a Baermann funnel were used for RNA extraction. For the RNA extractions, 10,000 nematodes were frozen in a mortar with liquid nitrogen and then powdered by using a pestle. The total RNA was extracted from the powder using TRIzol reagent (Invitrogen, USA, catalogue number 15596-026; Wang et al., 2012, 2016) followed by application of the Promega AMV reverse transcription system (catalogue number S3500) according to the manufacturer's instructions. We then used Oligo (dT)_18_ as the primer to obtain the first chain cDNA. *Ab-tps1* (accession number KY661388, using primers Ab-tps1-cd-F and Ab-tps1-cd-R; Table S1), *Ab-tps2* (accession number KY661389, using primers Ab-tps2-cd-F and Ab-tps2-cd-R; Table S1), *Ab-ntre1* (accession number MF997580, using primers Ab-ntre1-cd-F and Ab-ntre1-cd-R; Table S1), *Ab-tre* (renamed *Ab-ntre2* in this study, accession number KY661390, using primers Ab-ntre2-cd-F and Ab-ntre2-cd-R; Table S1) and *Ab-atre* (accession number MF997581, using primers Ab-atre-cd-F and Ab-atre-cd-R; Table S1) were then cloned using the first chain cDNA as the template (Qiaoli et al., 2017).

### Analysis of transcript abundance

The transcript levels of *Ab-tps1*, *Ab-tps2*, *Ab-ntre1*, *Ab-ntre2* and *Ab-atre* under anaerobic conditions (25°C, 0 mg l^−1^ dissolved oxygen) were measured every day by RT-qPCR using a GoTaq 2-Step RT-qPCR System Kit (Promega, USA, catalogue number A6010) and a Stratagene Mx3000P qPCR system (Agilent, USA). The reaction systems were as follows: firstly, pre-denaturation at 95°C for 2 min; then 95°C for 30 s, 60°C for 1 min and 72°C for 30 s for 40 cycles; melting curves were set from 55 to 95°C (Fig. S4). A constitutively expressed gene, Ab-28s RNA, was used as an internal control (using primers Ab-28s RNA-Q-F and Ab-28s RNA-Q-R; Table S1; Zhong and Simons, 1999). *Ab-tps1-Q* (using primers Ab-tps1-Q-F and Ab-tps1-Q-R; Table S1), *Ab-tps2-Q* (using primers Ab-tps2-Q-F and Ab-tps2-Q-R; Table S1), *Ab-ntre1-Q* (using primers Ab-ntre1-Q-F and Ab-ntre1-Q-R; Table S1), *Ab-ntre2-Q* (using primers Ab-ntre2-Q-F and Ab-ntre2-Q-R; Table S1) and *Ab-atre-Q* (using primers Ab-atre-Q-F and Ab-atre-Q-R; Table S1) were used as reference genes. The normalization of data followed the instructions of the GoTaq 2-Step RT-qPCR System Kit, and the 2^−ΔΔ*C*_T_^ method was used to analyse the data (Livak and Schmittgen, 2001).

This nematode could survive in aerobic distilled water for about 6 days. For anoxybiosis stages (1, 2, 3, 4 and 5 days), the nematodes were soaked in anaerobic distilled water as the test and aerobic distilled water (25°C, 8.25 mg l^−1^ dissolved oxygen) as the control. For re-aeration stages (0, 25, 50, 75, 100 and 125 min), the anoxybiosis nematodes were soaked in aerobic distilled water after a 1-day anaerobic treatment (when the survival rate underwent no obvious change). The control groups consisted of nematodes treated with aerobic distilled water for the same amount of time. In these experiments, three separate biological replicates of each treatment were performed and each replicate was assessed three times. A paired-sample Student's *t*-test was used to determine the difference between anaerobic-treated and aerobic-treated nematodes.

### RNA interference

RNA interference (RNAi) was performed using nematodes at mixed developmental stages, as described by Urwin et al. (Urwin et al., 2002; see also Wang et al., 2012; Qiaoli et al., 2017). Double-stranded RNAs (dsRNA) corresponding to *Ab-tps1* (using two primer pairs Ab-T7-tps1-F and Ab-tps1-iR/Ab-tps1-iF and Ab-T7-tps1-R, Table S1), *Ab-tps2* (using two primer pairs Ab-T7-tps2-F and Ab-tps2-iR/Ab-tps2-iF and Ab-T7-tps2-R, Table S1), *Ab-ntre1* (using two primer pairs Ab-T7-ntre1-F and Ab-ntre1-iR/Ab-ntre1-iF and Ab-T7-ntre1-R, Table S1), *Ab-ntre2* (using two primer pairs Ab-T7-ntre2-F and Ab-ntre2-iR/Ab-ntre2-iF and Ab-T7-ntre2-R, Table S1) and *Ab-atre* (using two primer pairs Ab-T7-atre-F and Ab-atre-iR/Ab-atre-iF and Ab-T7-atre-R, Table S1) were prepared using the MAXIscript T7/T3 RNA Synthesis Kit (Ambion, Japan, catalogue number AM1324). The *Ab-tps1* RNAi, *Ab-tps2* RNAi, *Ab-ntre1* RNAi, *Ab-ntre2* RNAi, *Ab-atre* RNAi, *Ab-tps1*+*Ab-tps1* RNAi, *Ab-ntre1*+*Ab-ntre2* RNAi, *Ab-ntre1+Ab-atre* RNAi, *Ab-ntre2+Ab-atre* RNAi and *Ab-ntre1*+*Ab-ntre2+Ab-atre* RNAi treated nematodes were soaked in M9 buffer with 10 mmol l^−1^ octopamine and the matching dsRNAs (3 mg ml^−1^), respectively. The CK (control check) nematodes were soaked in M9 buffer with 10 mmol l^−1^ octopamine only. After soaking for 12 h at 25°C with intermittent stirring, all the RNAi-treated nematodes were thoroughly washed with sterile water to remove the external dsRNA.

The RNAi-treated and CK nematodes were then divided into five groups. In order to determine the extent of RNAi, RT-qPCR experiments were performed by taking approximately 10,000 nematodes from each group to measure the transcript levels of *Ab-tps1*, *Ab-tps2*, *Ab-ntre1*, *Ab-ntre2* and *Ab-atre* (Ab-28s RNA was used as the internal control). In addition, approximately 50,000 nematodes were taken to assess the AT activity or NT activity, and another approximately 50,000 nematodes were taken to assess the trehalose level of each group, respectively. For different RNAi-treated nematodes, approximately 5000 nematodes were used to assess the survival of aerobic RNAi-treated nematodes and another approximately 5000 nematodes were used to assess the recovery of anaerobic RNAi-treated nematodes. For CK nematodes, 5000 nematodes were used to assess the survival of aerobic RNAi-free nematodes. Another 5000 nematodes were used to assess the recovery of anaerobic RNAi-free nematodes.

Anaerobic nematodes were cultured in anaerobic distilled water for 1–6 days, and aerobic nematodes were cultured in aerobic distilled water for the same amount of time. The recovery of anaerobic nematodes and the survival of aerobic nematodes in aerobic distilled water was monitored daily.

As a control, we investigated the gene silencing efficacy for *Ab-lea* (late embryogenesis abundant protein, LEA) and *Ab-ace* (acetylcholinesterase, ACE). LEA is associated with stress resistance, and has similar functions to trehalose metabolism genes; ACE is thought to play a role in the conduction of nerves, unlike TRE, TPP or LEA. Corresponding dsRNAs were prepared using two primer pairs Ab-T7-lea-F and Ab-lea-iR/Ab-lea-iF and Ab-T7-lea-R; and two primer pairs Ab-T7-ace-F and Ab-ace-iR/Ab-ace-iF and Ab-T7-ace-R (Table S1) with a MAXIscript T7/T3 RNA Synthesis Kit, respectively. RNAi, RT-qPCR (using primers Ab-lea-Q-F and Ab-lea-Q-R/Ab-ace-Q-F and Ab-ace-Q-R; Table S1) and survival assessment experiments were then performed as described previously to investigate gene silencing efficacy after RNAi treatment.

In these experiments, three separate biological replicates of each treatment were performed and each replicate was repeated three times. A paired-sample Student's *t*-test was used to determine the difference between RNAi-treated nematodes and CK nematodes, and a bivariate correlation analysis (SPSS 13.0) was used to determine how different gene-silencing stratagems influenced the recovery of nematodes from anoxybiosis.

### TRE activity and trehalose level determination

TRE activity of nematodes was determined based on the 3.5-dinitrosalicylic acid method. Reducing sugars were produced by TRE catalysing the hydration of trehalase. Reducing sugars and 3.5-dinitrosalicylic acid heated together will generate brownish-red amino compounds. In a certain range, the colour depth of the reaction fluid is in direct proportion to the level of the activity of TRE. These reactions were performed using the TRE Determination Kit (acidic version/neutral version; Cominbio, China, catalogue number HTM-2-Y) and a BCA Method of Protein Content Kit (Cominbio, catalogue number BCAP-2-W). According to the manufacturer's instructions, to measure the activity of NT, the neutral version (pH 7.0) of the TRE Determination Kit was used (Londesborough and Varimo, 1984); and to measure the activity of AT, the acidic version (pH 5.0) of the TRE Determination Kit was used (Dmitryjuk and Zółtowska, 2003).

A 0.5 mol l^−1^ trichloroacetic acid (TCA) solution was used to extract and guarantee that only trehalose was collected, rather than other sugars. Based on the anthrone colorimetry method, trehalose levels were then measured using the Trehalose Content Kit (Cominbio, catalogue number HT-2-Y) and BCA Method of Protein Content Kit.

Nematodes used for each test were part of the sample used for the RT-qPCR experiments under anoxybiosis or re-aeration. For each test, a GeneQuant 1300 ultraviolet spectrophotometer was used to measure the colour depth of the reaction fluids (Qiaoli et al., 2017). Three separate biological replicates of each treatment were performed and each replicate was assessed three times. A paired-sample Student's *t*-test was used to determine the differences between each time point.

## RESULTS

### Homologous TPS and TRE gene selection

There are five putative TRE-encoding genes identified in *C. elegans* (*tre1* encoding TRE1, *tre2* encoding TRE2, *tre3* encoding TRE3, *tre4* encoding TRE4 and *tre5* encoding TRE5) and two putative TPS-encoding genes (*tps1* encoding TPS1 and *tps2* encoding TPS2). Homologous TPS and TRE genes of *A. besseyi* from its transcriptome were identified using tblastn. *Ab-tps1* was searched based on TPS1; *Ab-tps2* was searched based on TPS2; *Ab-ntre1* was searched based on TRE1; *Ab-ntre2* was searched based on TRE2; and *Ab-atre* was searched based on TRE5. The matched sequences of TRE3 and TRE4 were not obtained.

The TPSs encoded by *Ab-tps1* and *Ab-tps2* are hydrophilic proteins, are located in cytoplasm and have no signal peptide. The TREs encoded by *Ab-ntre1* and *Ab-ntre2* are hydrophilic proteins, are located in cytoplasm and have no signal peptide; they are NTs. The TRE encoded by *Ab-atre* is a hydrophilic protein, is located in cytoplasm and has a signal peptide; it is an AT (Fig. S1). Hence, two TPS-encoding genes and three TRE (two NT and one AT)-encoding genes were identified in total.

### Analysis of transcript abundance

**Fig. 1.**
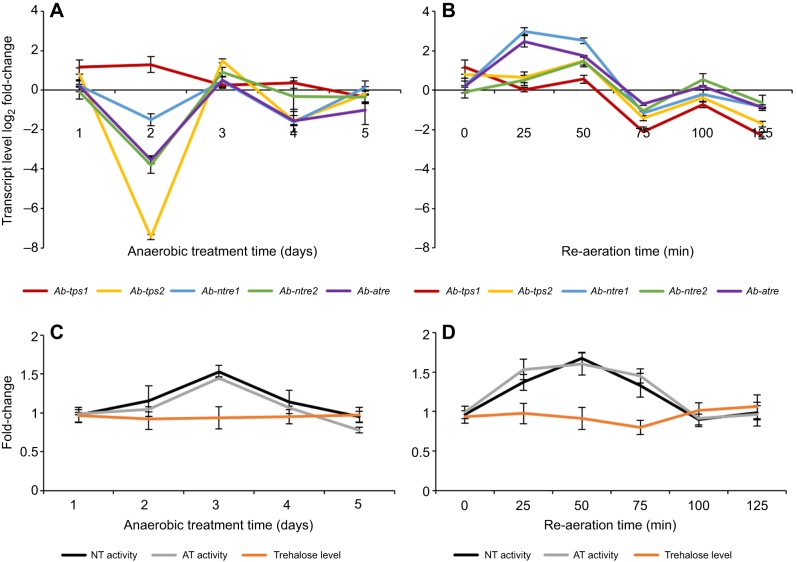
**Analysis of transcript relative abundance, TRE activity and trehalose levels for *Aphelenchoidesbesseyi* during anoxybiosis or during recovery after being kept under anoxybiosis for 1** **day**. (A) Transcript relative abundance of trehalose metabolism genes during anoxybiosis. (B) Transcript relative abundance of trehalose metabolism genes during recovery after being kept under anoxybiosis for 1 day. (C) AT activity, NT activity and trehalose level during anoxybiosis. (D) AT activity, NT activity and trehalose level during recovery after being kept under anoxybiosis for 1 day. Data are given as means with s.d. (*N*=3).

For the anoxybiosis stage, the transcript level of *Ab-tps1* increased at 1 and 2 days, but showed no difference from the control group at 3, 4 and 5 days. The transcript level of *Ab-tps2* showed no difference from the control group at 1 and 5 days, increased significantly at 3 days, but decreased significantly at 2 days and slightly at 4 days. The transcript level of *Ab-ntre1* showed no difference from the control group at 1, 3 and 5 days, but decreased significantly at 2 and 4 days. The transcript level of *Ab-ntre2* showed no difference from the control group at 1, 4 and 5 days, decreased significantly at 2 days, and increased slightly at 3 days. The transcript level of *Ab-atre* showed no difference from the control group at 1 and 3 days, decreased significantly at 2 and 4 days, and decreased slightly at 5 days (Fig. 1A, Fig. S2).

It was interesting to note that during anoxybiosis the increase of the transcript level of *Ab-tps2* and *Ab-ntre2* only happened at 3 days, when the transcript level of *Ab-tps1* began to decrease. No distinct increase of the transcript level of *Ab-ntre1* or *Ab-atre* was found. However, on the whole, variation trends of transcript levels of *Ab-tps1*, *Ab-ntre1*, *Ab-ntre2* and *Ab-atre* were similar; *Ab-tps2* had an opposite trend (Fig. 1A, Fig. S2).

For the re-aeration stage, the transcript level of *Ab-tps1* showed no difference from the control group at 0 min to 50 and 100 min, but decreased significantly at 75 and 125 min. The transcript level of *Ab-tps2* did not change obviously when nematodes were under re-aeration for 0 to 25 min, as well as for 75 and 100 min; however, levels increased significantly at 50 min and decreased significantly at 125 min. The transcript level of *Ab-ntre1* rose significantly when nematodes were under re-aeration for 0 to 50 min, but then declined at 75 min and was similar to the control group at 100 and 125 min. The transcript level of *Ab-ntre2* did not change obviously when nematodes were under re-aeration for 0 to 25 min, as well as for 75 and 125 min; however, levels increased significantly at 50 min. There was an increase in the level of *Ab-atre* transcript as the time of re-aeration increased from 0 to 50 min, but this appeared to decrease and became similar to the control group for 75 to 125 min (Fig. 1B, Fig. S2).

It was also interesting to note that the increase in transcript level of *Ab-tps2* and *Ab-ntre2* only occurred at 50 min. The increase of the transcript level of *Ab-ntre1* and *Ab-atre* occurred at 25 and 50 min. No distinct increase of the transcript level of *Ab-tps1* was found after re-aeration. Nevertheless, the basic tendency of changes was the same for all five genes (Fig. 1B), which was quite different compared with the anoxybiosis stage.

### Changes in TRE activity and trehalose level during anoxybiosis and re-aeration

It was not surprising to find that changing trends for NT activity and AT activity were similar based on similar trends of their encoding genes. During anoxybiosis, after soaking in an anaerobic distilled water for 1 and 2 days, NT activity and AT activity were both similar to the control group. They both increased at 3 days and then fell at 4 and 5 days (Fig. 1C, Fig. S3). During re-aeration periods, there were increases in both NT activity and AT activity as the time of re-aeration increased from 0 to 75 min; the highest levels occurred at 50 min. However, NT activity and AT activity both decreased to be similar to the control group at 100 and 125 min (Fig. 1D, Fig. S3).

Compared with the control group, when the nematodes were undergoing anoxybiosis, their trehalose levels were lower than the control group (but not significantly), and then rose slightly over time (Fig. 1C, Fig. S3). During re-aeration, their trehalose levels decreased when the time of re-aeration increased from 50 to 75 min, but they were similar to the control group for the other time points (Fig. 1D, Fig. S3). The changes of trehalose levels matched the changes of TRE activity (Fig. 1C,D, Fig. S3). However, trehalose levels should be influenced by both *Ab-tps1* and *Ab-tps2*. Therefore, the fact that trehalose levels did not alter significantly during anaerobic treatment might be due to contrary changes of transcript levels of *Ab-tps1* and *Ab-tps2*.

### RNAi

Significant silencing was found, along with reasonable changes in NT activity, AT activity and trehalose level (Table 1). Significant silencing was also found for *Ab-lea* and *Ab-ace* after RNAi treatment (log_2_RNAi/CK fold*_Ab-lea_*=−1.58±0.51; log_2_RNAi/CK fold*_Ab-ace_*=−1.67±0.02), which indicated that RNAi by soaking was potent for *A. besseyi*. The silencing of one TPS gene would reduce the transcript level of the other TPS gene and also lessened the level of trehalose as a direct consequence. However, if both TPS genes were silenced, then transcript levels of *Ab-ntre1*, *Ab-ntre2* and *Ab-atre* would decrease. Silencing of *Ab-ntre1*, *Ab-ntre2* or *Ab-atre* would also decrease transcript levels of both TPS genes. As a result, TRE activity would decrease and trehalose levels would increase (Table 1).

**Table 1.**
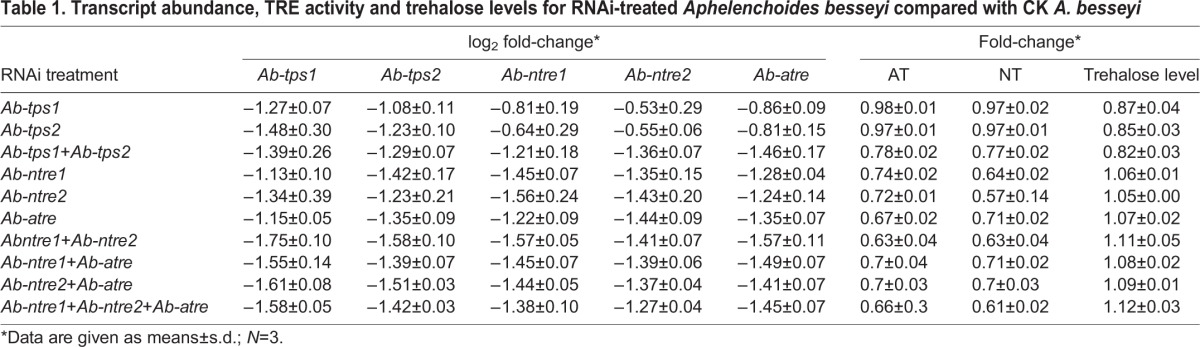
**Transcript abundance, TRE activity and trehalose levels for RNAi-treated *Aphelenchoidesbesseyi* compared with CK *A. besseyi***

**Fig. 2.**
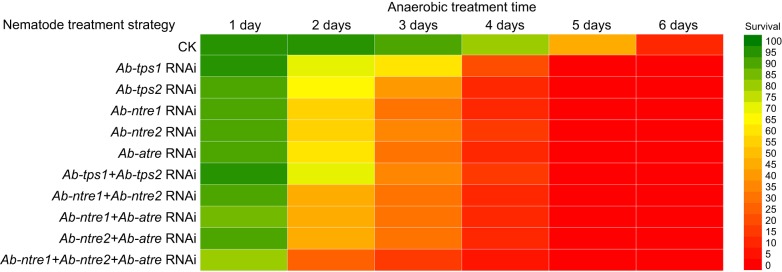
**Survival comparison of differently treated *A. besseyi* under anaerobic conditions.** Data are given as means (*N*=3).

**Fig. 3.**
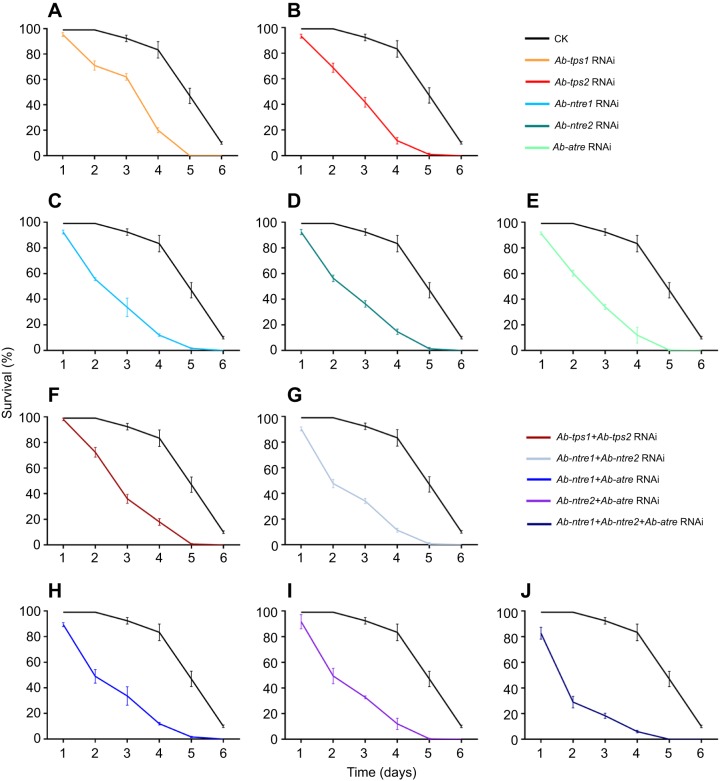
**Survival of differently treated *A. besseyi* under anaerobic conditions.** Lines indicate differently treated nematodes. Data are given as means with s.d. (*N*=3). (A–J) Survival of anaerobic nematodes treated with CK and the following RNAi: (A) *Ab-tps1* RNAi, (B) *Ab-tps2* RNAi, (C) *Ab-ntre1* RNAi, (D) *Ab-ntre2* RNAi, (E) *Ab-atre2* RNAi, (F) *Ab-tps1+Ab-tps-2* RNAi, (G) *Ab-ntre1+Ab-ntre2* RNAi, (H) *Ab-ntre1+Ab-atre* RNAi, (I) *Ab-ntre2+Ab-atre* RNAi and (J) *Ab-ntre1+Ab-ntre2+Ab-atre* RNAi.

All the aerobic RNAi-treated nematodes presented a survival rate of more than 99% for the 6 days of the study, which was not significantly different from that of the control group. There was no distinct difference in survival rate and morphology between RNAi-treated nematodes and CK nematodes when they were all under anaerobic conditions for 0 min to 1 day; however, all the anaerobic RNAi-treated groups showed regular and significantly downward survival trends for 2 to 6 days of anaerobic treatment (Figs 2 and 3).

The survival of anaerobic *Ab-tps1* RNAi-treated nematodes decreased to approximately 70% at 2 days and continued to decrease to approximately 60% at 3 days, 20% at 4 days and 0% at 5 and 6 days. The survival of anaerobic *Ab-tps2* RNAi-treated nematodes also decreased to approximately 70% at 2 days and continued to decrease to approximately 40% at 3 days, 10% at 4 days, 1% at 5 days and 0% at 6 days. The survival of anaerobic *Ab-ntre1* RNAi-treated nematodes decreased to approximately 60% at 2 days and continued to decrease to approximately 35% at 3 days, 10% at 4 days, 2% at 5 days and 0% at 6 days. The survival of anaerobic *Ab-ntre2* RNAi-treated nematodes decreased to approximately 60% at 2 days and continued to decrease to approximately 35% at 3 days, 15% at 4 days, 1% at 5 days and 0% at 6 days. The survival of anaerobic *Ab-atre* RNAi-treated nematodes decreased to approximately 60% at 2 days and continued to decrease to approximately 35% at 3 days, 10% at 4 days and 0% at 5 and 6 days (Figs 2 and 3A–E). The results of RT-qPCR showed that the transcript level of *Ab-tps1* increased only when anaerobic-treated for 1 and 2 days, and the change was opposite to that for *Ab-tps2*, *Ab-ntre1*, *Ab-ntre2* or *Ab-atre* (Fig. 1). These combined results might account for the different survival rates observed at 3 days for *Ab-tps1* RNAi-treated anaerobic nematodes (Figs 2 and 3).

The survival of *Ab-tps1+Ab-tps2* RNAi-treated nematodes was similar to that of *Ab-tps2* RNAi-treated or *Ab-ntre2* RNAi-treated nematodes, which was approximately 70% at 2 days and decreasing to approximately 35% at 3 days, 20% at 4 days, 1% at 5 days and 0% at 6 days. The survival of *Ab-ntre1+Ab-ntre2* RNAi-treated nematodes was approximately 50% at 2 days, 35% at 3 days, 10% at 4 days, 1% at 5 days and 0% at 6 days. The survival of *Ab-ntre1+Ab-atre* RNAi-treated nematodes was approximately 50% at 2 days, 30% at 3 days, 10% at 4 days, 2% at 5 days and 0% at 6 days. The survival of *Ab-ntre2+Ab-atre* RNAi-treated nematodes was approximately 50% at 2 days, 30% at 3 days, 10% at 4 days and 0% at 5 and 6 days. The survival of *Ab-ntre1+Ab-ntre2+Ab-atre* RNAi-treated nematodes was approximately 30% at 2 days, 20% at 3 days, 5% at 4 days and 0% at 5 and 6 days (Figs 2 and 3F–J).

However, the survival of RNAi-free nematodes under anaerobic conditions for 1–3 days was approximately 90–99%, which was significantly higher than that of RNAi-treated groups (Figs 2 and 3). Although survival of the RNAi-free nematodes declined to approximately 80% at 4 days, 50% at 5 days and 10% at 6 days, this was still dramatically higher than that of RNAi-treated groups (Figs 2 and 3). Student's *t*-test results indicated that the survival rate of RNAi-treated groups differed significantly from that of the CK group (Table S2).

**Fig. 4.**
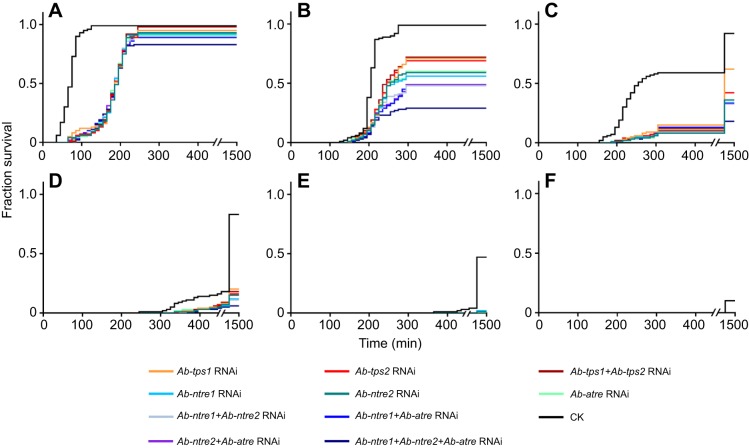
**Recovery of differently treated *A. besseyi*.** Recoveries are shown for: (A) 1 day anaerobic-treated *A. besseyi*; (B) 2 days anaerobic-treated *A. besseyi*; (C) 3 days anaerobic-treated *A. besseyi*; (D) 4 days anaerobic-treated *A. besseyi*; (E) 5 days anaerobic-treated *A. besseyi*; and (F) 6 days anaerobic-treated *A. besseyi*. Lines indicated differently treated nematodes. Data are given as means (*N*=3).

The bivariate correlation analysis between survival rates and time of anaerobic treatment for RNAi-treated groups showed significant correlations (*r_tps1_*=−0.97, *r_tps2_*=−0.97, *r_ntre1_*=−0.95, *r_ntre2_*=−0.96, *r_atre_*=−0.96, *r_tps1+tps2_*=−0.97, *r_ntre1+ntre2_*=−0.94, *r_ntre1+atre_*=−0.95, *r_ntre2+atre_*=−0.94 and *r_ntre1+ntre2+atre_*=−0.87), as well as for the CK group (*r*_CK_=−0.91). This indicated that there was a correlation between survival rate and anaerobic treatment time. However, the recovery time of RNAi-treated nematodes was much longer than that of CK nematodes (Fig. 3) and during re-aeration the recovery of RNAi-treated nematodes was delayed compared with that of CK nematodes (Fig. 4).

## DISCUSSION

To date, accumulation of trehalose or over-expression of TPS genes and TRE genes upon desiccation (Madin and Crowe, 1975; Adhikari et al., 2009) or hypertonic osmotic pressure (Qiaoli et al., 2017) has been reported in numerous nematodes. However, little has been reported on the mechanism of trehalose metabolism in nematodes under anoxic stress (Qiu and Bedding, 2000). Our study indicates that the upregulation of the transcript level of *Ab-tps1* only occurred when *A. besseyi* was soaked in anaerobic distilled water for 1 to 2 days; upregulation of the transcript level of *Ab-tps2* only occurred when *A. besseyi* was soaked in anaerobic distilled water for 3 days or under re-aeration for 50 min; upregulation of transcript levels of *Ab-ntre1* and *Ab-atre* occurred when *A. besseyi* was under re-aeration for 25 or 50 min; finally, upregulation of the transcript level of *Ab-ntre2* only occurred when *A. besseyi* was under re-aeration for 50 min. These observations revealed that *Ab-tps1* and *Ab-tps2* were upregulated in *A. besseyi* only during certain periods of anoxybiosis, and *Ab-tps2*, *Ab-ntre1*, *Ab-ntre2* and *Ab-atre* were upregulated in *A. besseyi* at a certain point of recovery.

Trehalose metabolism has been well characterized in many plants and microorganisms. Evidence has indicated that it is sucrose instead of trehalose produced by plants that primarily acts as a bioprotectant (Delorge et al., 2014; Lunn et al., 2014). However, it has also been highlighted repeatedly that TPS and TPP play important roles in regulating plant metabolism, growth, development and abiotic stress response (Li et al., 2011; Ponnu et al., 2011; Lunn et al., 2014; Tsai and Gazzarrini, 2014). Moreover, although most of the bdelloid rotifers appear to be desiccation resistant, trehalose has never been detected in them (Hespeels et al., 2015). However, trehalose might be involved in osmotic regulation of a monogonont species (Caprioli et al., 2004). In addition, at each time point, higher expression levels for TRE genes than for TPS genes have been observed in the bdelloid rotifer *Adineta vaga* during the drying–rehydration process, which might explain why trehalose was undetected previously (Hespeels et al., 2015). In numerous desiccation-resistant nematodes, accumulation of trehalose or over-expression of TPS genes upon desiccation was reported (Madin and Crowe, 1975; Adhikari et al., 2009). Our previous investigation indicated that the changes in NT activity were consistent with the transcript level changes of *Ab-ntre2* (formerly named *Ab-tre*) when *A. besseyi* was in (as well as recovering from) osmobiosis, and the trehalose levels declined at certain periods when the TRE activity was enhanced, suggesting that the hydrolysis of trehalose is essential under hypertonic osmotic pressure (Qiaoli et al., 2017). This previous study also indicated that during the dehydration–rehydration process, the changes in transcript levels of *Ab-tps1*, *Ab-tps2* and *Ab-tre* followed similar trends (Qiaoli et al., 2017).

In this study, however, we observed that this circumstance happened only during the re-aeration period. It was interesting to note that the changes in transcript level of *Ab-tps1* was opposite to that for *Ab-tps2*, *Ab-ntre1*, *Ab-ntre2* or *Ab-atre* during anoxybiosis (Fig. 1, Fig. S2). Nevertheless, on the whole, transcript levels of *Ab-ntre1*, *Ab-ntre2* and *Ab-atre* were commensurate with combined transcript levels of *Ab-tps1* and *Ab-tps2* (Fig. 1A, Fig. S2). These results suggest that *Ab-tps1* and *Ab-tps2* might function together when the nematodes are in anoxybiosis, to accumulate levels of trehalose and affect the transcript level of *Ab-ntre1*, *Ab-ntre2* and *Ab-atre*. *Ab-tps1* and *Ab-tps2* might also function during recovery from anoxybiosis to facilitate the accumulation of trehalose, and *Ab-ntre1*, *Ab-ntre2* and *Ab-atre* might function at the same time in order to promote the hydrolysation of trehalose (Fig. 1B, Fig. S2). However, during this period, the transcript level of *Ab-tps1* was lower at every time point compared with that of *Ab-tps2* (Fig. 1B). This result indicates that transcript patterns of *Ab-tps1*, *Ab-tps2*, *Ab-ntre1*, *Ab-ntre2* and *Ab-atre* were not all the same, which was distinct from our earlier study (Qiaoli et al., 2017). This situation may have occurred because those genes react differentially to various environmental stresses.

TRE was classified into AT and NT according to their pH optima; they are essential for the utilization of extracellular trehalose and the mobilization of intracellular trehalose, respectively (Londesborough and Varimo, 1984; Basu et al., 2006). Our investigation suggested that variation trends of transcript levels of *Ab-tps1*, *Ab-ntre1*, *Ab-ntre2* and *Ab-atre* were similar when the nematode was under anaerobic and re-aeration conditions (Fig. 1A,B). It was therefore not surprising to discover that the changing trends for NT activity and AT activity were similar based on the similar trends of their encoding genes (Fig. 1C,D). The consumption of trehalose may be catalysed by both AT and NT at the same time, and to survive anaerobic environments the nematode may utilize both extracellular trehalose and intracellular trehalose.

It has been reported that under anaerobic conditions, the trehalose content of *S. carpocapsae* decreased sharply while lipid and protein contents did not change substantially (Qiu and Bedding, 2000). When anaerobically incubated *S. carpocapsae* were returned to an aerobic environment, both glycogen and trehalose levels increased while lipid levels decreased sharply (Qiu and Bedding, 2000). In our investigation, the observation was a little different. During anoxybiosis, changes in TRE activity were consistent with changes in the transcript level of *Ab-ntre1*, *Ab-ntre2* and *Ab-atre*, with an obvious increase appearing at 3 days; however, the trehalose levels continued to be a little lower than the control nematodes with a slightly increasing trend (Fig. 1A,C). We also noticed that for 1 to 2 days, the TRE activity and the trehalose level of treated nematodes were both steady and similar to those of CK nematodes (Fig. 1C). This was unexpected as transcript levels of *Ab-tps2*, *Ab-ntre1*, *Ab-ntre2* and *Ab-atre* were very low but the transcript level of *Ab-tps1* was obviously upregulated (Fig. 1A). However, when nematodes were treated for 3 days, transcript levels of *Ab-ntre1*, *Ab-ntre2* and *Ab-atre* were upregulated significantly compared with 2 days, and that of *Ab-tps2* increased even more (Fig. 1A). Also, we could not ignore the fact that the transcript level of *Ab-tps1* was exactly the opposite; it decreased to be similar to that of CK nematodes (Fig. 1A). As a result, the TRE activity increased dramatically but the trehalose level remained steady (Fig. 1C).

This biological pattern was similar to trehalose metabolism pathways observed in bdelloid rotifers and plants, where the TPS and TRE genes may significantly change at different time points and the TRE activity is so high that the over-expression of TPS genes does not result in an increase in trehalose level (Fig. 1C; Müller et al., 2001; Lunn et al., 2014; Hespeels et al., 2015). However, obviously upregulated transcript levels of of *Ab-tps1* and *Ab-tps2* were observed for 1 day to 2 and 3 days (Fig. 1A), respectively, suggesting a possible signalling role of trehalose in this process (Hespeels et al., 2015). It has been reported that as plants have many signalling molecules, in order to prevent the accumulation of trehalose from interfering with the regulation of plant metabolism, a rapid degradation of it may therefore be required (Wingler, 2002; Fernandez et al., 2010). This might account for the steady low level of trehalose during anoxybiosis (Fig. 1C) and the extreme decrease of *Ab-tps2* at 2 days (Fig. 1A).

During re-aeration, the transcript level of *Ab-tps2* increased, whereas the transcript level of *Ab-tps1* showed no obvious change compared with the control nematodes (Fig. 1B). The changes in TRE activity were consistent with the changes in the transcript levels of *Ab-ntre1*, *Ab-ntre2* and *Ab-atre*. This resulted in a low level of trehalose when the nematodes were under re-aeration for 75 min after suffering anaerobic treatment for 1 day (Fig. 1C), and could be attributed to sharp increases in transcript levels of *Ab-ntre1*, *Ab-ntre2* and *Ab-atre* at 50 min (Fig. 1B). These observations also suggest that the accumulation and consumption of trehalose may be synchronized and regulated by multiple genes together at certain periods. As a result, the consumption of trehalose could provide various tissues and organs with glucose, and could effectively protect cells in the nematode by adaption to oxygen limitation, thereby enhancing their resilience (Behm, 1997).

**Fig. 5.**
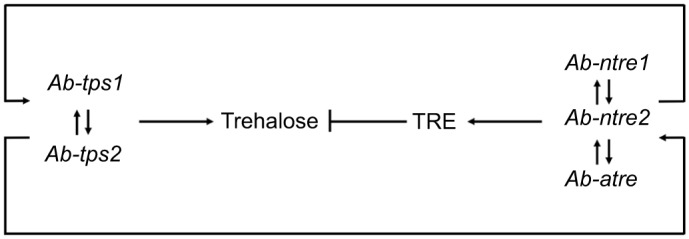
**Regulation between trehalose metabolism genes, TRE activity and trehalose levels.**

Thus, considering changes in trehalose levels, we assume that trehalose in *A. besseyi* could act as an energy supply source as well as a signal molecule, and its metabolism genes may work together to control its levels (Fig. 5). Although it has been demonstrated that expression of trehalose metabolism genes may be different under different environmental stimuli, we also observed similar trends of trehalose levels between nematodes under hypertonic osmotic pressure and anaerobic stress (Qiaoli et al., 2017). On the whole, trehalose levels were quite steady in spite of the drastic changes of TRE activity during osmobiosis or anoxybiosis (Qiaoli et al., 2017). These results may suggest that despite the different expression patterns of the trehalose metabolism genes, to maintain the survival of the nematode under different environmental stresses, the trehalose level and TRE activity may result in similar states (Fig. 5).

Results of RNAi treatments have shown that trehalose metabolism genes play important roles in helping *A.* *besseyi* to survive anaerobic treatment. If any of TPS or TRE genes were temporarily knocked down (individually or in groups) before the nematodes were under anaerobic conditions, the mortality of the nematodes would increase and recovery would be delayed (Figs 3 and 4). Also, compared with all the anaerobic RNAi-treated groups, the survival of *Ab-tps2* RNAi-treated, *Ab-ntre1* RNAi-treated, *Ab-ntre2* RNAi-treated and *Ab-atre* RNAi-treated nematodes were similar, although the survival of *Ab-tps1* RNAi-treated nematodes was a little higher at 2–4 days (Figs 2 and 3). It has been shown that *Ab-ntre1*, *Ab-ntre2* and *Ab-atre* had no obvious upregulation of transcript level during anoxybiosis and *Ab-tps1* had no obvious upregulation of transcript level during re-aeration (Fig. 1A,B). Considering survival results with changes of transcript levels of these genes, we might conclude that the genes functioning at re-aeration have a greater impact on nematode survival (Figs 2 and 3). This demonstrates the essential roles of trehalose metabolism genes in anoxybiosis survival.

When either TPS gene was silenced, the transcript level of the other TPS gene and the trehalose level decreased, but the AT activity and NT activity did not change. Nevertheless, when *Ab-tps1* and *Ab-tps2* were both silenced, both AT activity and NT activity decreased. In addition, when one TRE-encoding gene was silenced, the transcript levels of both TPS genes and both AT activity and NT activity decreased, whereas the trehalose level increased (Table 1). These results indicate that there may be a feedback regulation mechanism between the trehalose metabolism genes. Each TPS gene and TRE gene may regulate each other, while each TRE gene may also regulate both TPS genes; and two TPS genes together may affect TRE genes (Fig. 5). This result was the same as in our previous study (Qiaoli et al., 2017). Our former investigation also indicated that survival was reduced for nematodes treated with genes silenced simultaneously (Qiaoli et al., 2017). Due to the different roles trehalose metabolism genes play in abiotic stress, we found in this study that the *Ab-tps1*+*Ab-tps2* RNAi treatment did not lead to a more significant decrease in survival, and the survival of nematodes undergoing *Ab-tps1* RNAi treatment was higher than for those undergoing other RNAi treatments (Figs 2 and 3). Thus diverse environmental stresses may result in differing expressions of trehalose metabolism genes.

To date, no TPP gene or domain has been identified in nematodes; it is possible that nematodes may synthesize endogenous trehalose using unspecific phosphatases (Sengupta et al., 2011a,b; Hespeels et al., 2015). Trehalose is suggested to be a signalling molecule, as well as a bioprotective molecule (Hespeels et al., 2015). Moreover, the synthesis of trehalose-6-phosphate (T6P), which is the trahalose precursor, has been shown to play a possible signalling role in plant, yeast and bdelloid rotifer (Schluepmann et al., 2003; Paul et al., 2008; Sengupta et al., 2011a,b; Hespeels et al., 2015), which might account for the low trehalose levels. There is also growing evidence that it is more likely that one of its precursors or enzymes involved in its synthesis instead of trehalose itself that serves as a signalling molecule controlling certain metabolic pathways (Avonce and Leyman, 2004; Yadav et al., 2014). To confirm whether these hypotheses are true for *A. besseyi* will require more appropriate metabolites and enzyme activities. However, the investigation of the roles played by TPS and TRE genes in *A. besseyi* during anoxybiosis and re-aeration indicated that trehalose metabolism genes play essential roles in protecting nematodes against an anaerobic environment. In spite of different expression patterns of trehalose metabolism genes, trehalose levels and TRE activity may result in similar states under different environmental stresses (Qiaoli et al., 2017). In addition, trehalose in *A.* *besseyi* may act as a signal molecule as well as an energy supply source, and its metabolism genes may work together to control its level (Fig. 5).

### Conclusion

Diverse environmental stresses may result in differing expressions of trehalose metabolism genes; however, trehalose level and TRE activity may result in similar states under different environmental stresses. The upregulation of the transcript levels of TPS and TRE genes occurs within certain periods of the anoxybiosis–re-aeration process. Each TPS gene may positively regulate the other, and each TRE gene may positively regulate both TPS genes and vice versa (Fig. 5). If the nematode was under anaerobic conditions after any of TPS genes and TRE genes were temporarily knocked down (individually or in groups), the mortality of the nematodes would increase and the recovery would be delayed (Figs 3 and 4). In addition, genes functioning at re-aeration have a greater impact on nematode survival under anaerobic conditions. The consumption of trehalose may catalysed by both AT and NT at the same time. Hence to survive an anaerobic environment, the nematode may utilize both extracellular trehalose and intracellular trehalose. Trehalose in *A.* *besseyi* may act as a signal molecule as well as an energy supply source, and its metabolism genes may work together to control its level (Fig. 5). Therefore, agents that are able to disable any of these genes could be useful to control this nematode.

## Supplementary Material

Supplementary information
