# Supplementary material for: Trehalose metabolism genes render rice white tip nematode Aphelenchoides besseyi (Nematoda: Aphelenchoididae) resistant to an anaerobic environment
Source: J Exp Biol. 2018 Feb 15;221(4):jeb171413. doi: 10.1242/jeb.171413 (PMC5868927; doi:10.1242/jeb.171413)
Supplement: Supplementary information [file jexbio-221-171413-s1.pdf]

**Table S1.** Primers used in this study

| Primer Name | Primer Sequence |
|---|---|
| Ab-tps1-cd-F | 5′-AGC ACA TGT ACG GTT TCG AG-3′ |
| Ab-tps1-cd-R | 5′-AGC GAT TGA CGA CAA GAT CC-3′ |
| Ab-tps1-Q-F | 5′-AGT GCA ATC ACC ATC ATC CG-3′ |
| Ab-tps1-Q-R | 5′-TGT TAC TCC ACG CAA AGA CG-3′ |
| Ab-T7-tps1-F | 5′-TAA TAC GAC TCA CTA TAG GGC AAT TCG TGG TCG ACA GAT CG-3′ |
| Ab-tps1-iR | 5′-GTC GAA CAT GCA AAA TGC GA-3′ |
| Ab-tps1-iF | 5′-AAT TCG TGG TCG ACA GAT CG-3′ |
| Ab-T7-tps1-R | 5′-TAA TAC GAC TCA CTA TAG GGC GTC GAA CAT GCA AAA TGC GA-3′ |
| Ab-tps2-cd-F | 5′-CAA CGA CGA CAA CAC CAA AG-3′ |
| Ab-tps2-cd-R | 5′-TTA ACA CGA TCC AAG GCG TC-3′ |
| Ab-tps2-Q-F | 5′-AGA GCA CGT ACA AGA GCA AC-3′ |
| Ab-tps2-Q-R | 5′-AAC ACA CTG ATC GCG TTC TT-3′ |
| Ab-T7-tps2-F | 5′-TAA TAC GAC TCA CTA TAG GGC TCT TCA GCC CTA TCA TCC GA-3′ |
| Ab-tps2-iR | 5′-AGG ACT TCC AAG AGG TCC AG-3′ |
| Ab-tps2-iF | 5′-TCT TCA GCC CTA TCA TCC GA-3′ |
| Ab-T7-tps2-R | 5′-TAA TAC GAC TCA CTA TAG GGC AGG ACT TCC AAG AGG TCC AG-3′ |
| Ab-atre-cd-F | 5′-TCA CAA CCA CCG CTA CTC AC-3′ |
| Ab-atre-cd-R | 5′-CCA AAT CCT GCT TGA ACC GC-3′ |
| Ab-atre-Q-F | 5′-GGC ATC CAC GTC TTG ATT CT-3′ |
| Ab-atre-Q-R | 5′-CCG ATG GAA AAT GTT CGC TG-3′ |
| Ab-T7-atre-F | 5′-TAA TAC GAC TCA CTA TAG GGC GCA CAA GAA CAC CGG ACA AC-3′ |
| Ab-atre-iR | 5′-CCA AAT CCT GCT TGA ACC GC-3′ |
| Ab-atre-iF | 5′-GCA CAA GAA CAC CGG ACA AC-3′ |
| Ab-T7-atre-R | 5′-TAA TAC GAC TCA CTA TAG GGC CCA AAT CCT GCT TGA ACC GC-3′ |
| Ab-ntre1-cd-F | 5′-CTG TAG TGG TCG TCT GCT GG-3′ |
| Ab-ntre1-cd-R | 5′-GCG ACC GAT TTC TCT TCC CA-3′ |
| Ab-ntre1-Q-F | 5′-CGG GGC TTC ATA ATA GGC AT-3′ |
| Ab-ntre1-Q-R | 5′-ATG CAG AGC GCA GTT AAT GT-3′ |
| Ab-T7-ntre1-F | 5′-TAA TAC GAC TCA CTA TAG GGC CAC AAC CAC CGC TAC TCA CT-3′ |
| Ab-ntre1-iR | 5′-ATT TCT CCA CGC GAC CGA TT-3′ |

| | |
|---|---|
| Ab-ntre1-iF | 5′-CAC AAC CAC CGC TAC TCA CT-3′ |
| Ab-T7-ntre1-R | 5′-TAA TAC GAC TCA CTA TAG GGC ATT TCT CCA CGC GAC CGA TT-3′ |
| Ab-ntre2-cd-F | 5′-CAA AGG TAT CTG GTT GGA TTT C-3′ |
| Ab-ntre2-cd-R | 5′-CAA TAA TAC GGG GAA TAG CAA C-3′ |
| Ab-ntre2-Q-F | 5′-ATG ATG TCA ACT GTT CGG GG-3′ |
| Ab-ntre2-Q-R | 5′-GAT CGT TTG TTG CGT CCA AG-3′ |
| Ab-T7-ntre2-F | 5′-TAA TAC GAC TCA CTA TAG GGC AAC CGT CAT CAC CAA CTG GA-3′ |
| Ab-ntre2-iR | 5′-ATC ATG CCC CGA ACA GTT GA-3′ |
| Ab-ntre2-iF | 5′-AAC CGT CAT CAC CAA CTG GA-3′ |
| Ab-T7-ntre2-R | 5′-TAA TAC GAC TCA CTA TAG GGC ATC ATG CCC CGA ACA GTT GA-3′ |
| Ab-28sRNA-F | 5′-TAC GAT CGG TGT TCG TTG C-3′ |
| Ab-28sRNA-R | 5′-CTC ACA TCG TCG ACA TCC AA-3′ |
| Ab-T7-lea-F | 5\`-TAA TAC GAC TCA CTA TAG GGC GGG TGC GAG CAA AGT GAA TG-3\` |
| Ab-lea-iR | 5\`-TCG GCA TGA CCC ATC ACA AA-3\` |
| Ab-lea-iF | 5\`-GGG TGC GAG CAA AGT GAA TG-3\` |
| Ab-T7-lea-R | 5\`-TAA TAC GAC TCA CTA TAG GGC TCG GCA TGA CCC ATC ACA AA-3\` |
| Ab-lea-Q-F | 5\`-CGT TCA CTG TGA TGA TGC CG-3\` |
| Ab-lea-Q-R | 5\`-AAA CCC AGC GGA GTT AGA CG-3\` |
| Ab-T7-ace-F | 5\`-TAA TAC GAC TCA CTA TAG GGC AGT CGT TGG GAA GGT GAA C-3\` |
| Ab-ace-iR | 5\`-AGT CGT TGG GAA GGT GAA C-3\` |
| Ab-ace-iF | 5\`-GGC TTC CCG CAA GAT AAC AG-3\` |
| Ab-T7-ace-R | 5\`-TAA TAC GAC TCA CTA TAG GGC GGC TTC CCG CAA GAT AAC AG-3\` |
| Ab-ace-Q-F | 5\`-GTT GGG TAT TGT ATG GTT CTC-3\` |
| Ab-ace-Q-R | 5\`-TGG TGA ATC AGC GGG TGC-3\` |

Journal of Experimental Biology • Supplementary information

**Table S2.** Significance of difference for the survival of *A. besseyi* between the RNAi-treated and RNAi-free *A. besseyi*.

| Treatment | Significance at *P* | *, *P*<0.05; **, *P*<0.01 |
|---|---|---|
| *Ab-tps1* RNAi | 0.010146945 | * |
| *Ab-tps2* RNAi | 0.008880087 | ** |
| *Ab-ntre1* RNAi | 0.006874695 | ** |
| *Ab-ntre2* RNAi | 0.006576599 | ** |
| *Ab-atre* RNAi | 0.006781937 | ** |
| *Ab-tps1+Ab-tps2* RNAi | 0.011269363 | * |
| *Ab-ntre1+Ab-ntre2* RNAi | 0.005855574 | ** |
| *Ab-ntre1+Ab-atre* RNAi | 0.005518363 | ** |
| *Ab-ntre2+Ab-atre* RNAi | 0.006391233 | ** |
| *Ab-ntre1+Ab-ntre2+Ab-atre* RNAi | 0.005987501 | ** |

Journal of Experimental Biology • Supplementary information

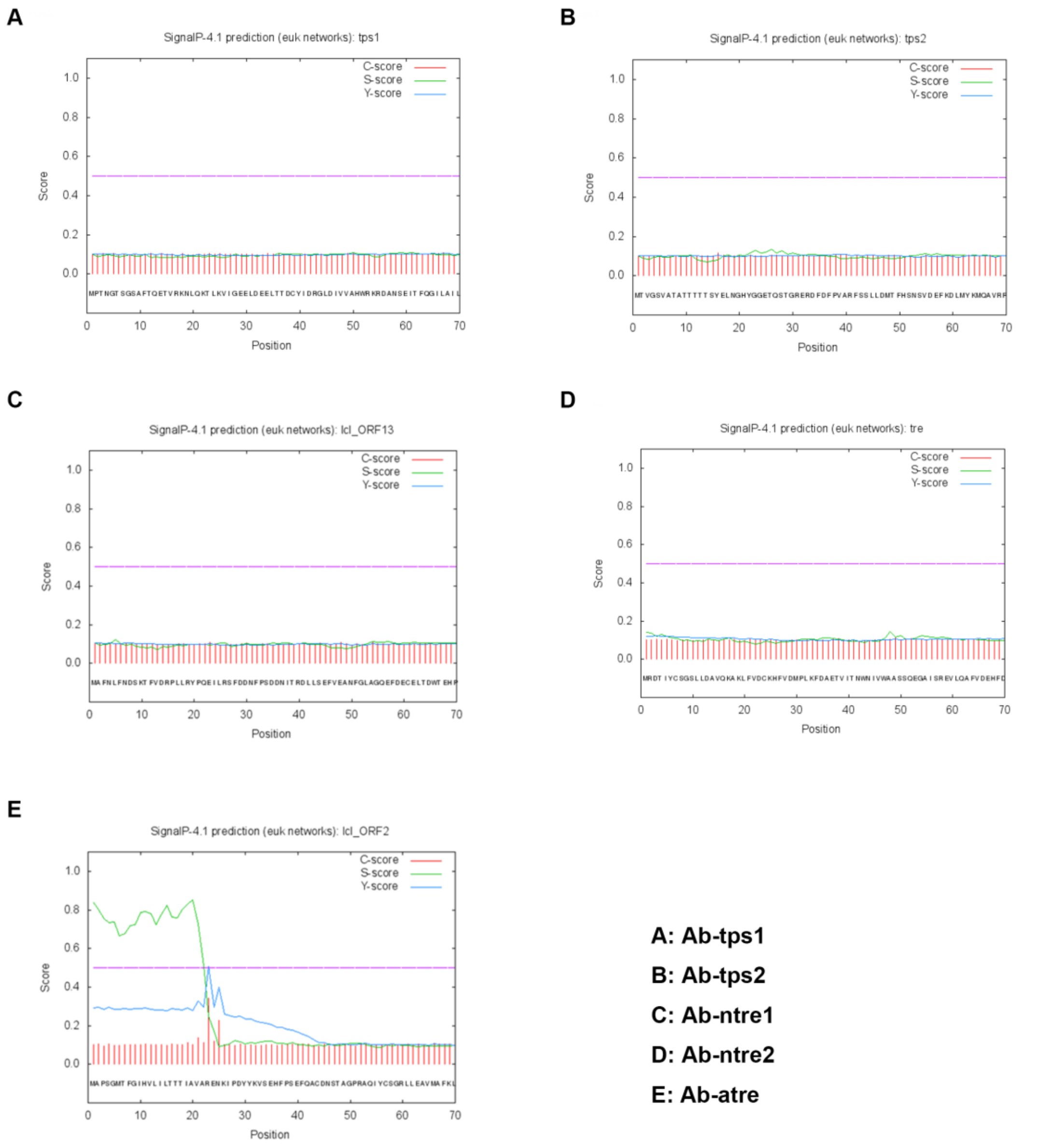


**Fig. S1. Signal peptide analysis for *Ab-tps1*, *Ab-tps2*, *Ab-ntre1*, *Ab-ntre2* and *Ab-atre* encoding proteins.**

Journal of Experimental Biology • Supplementary information

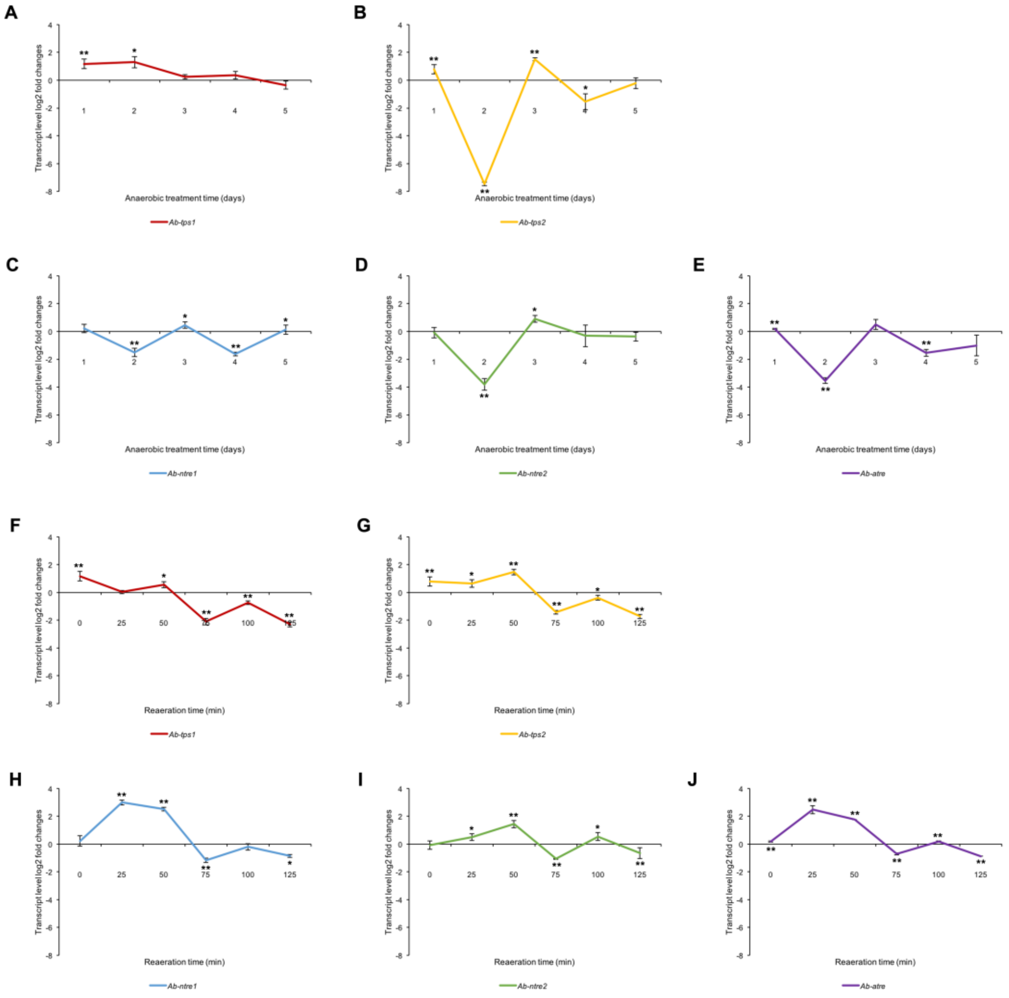


**Fig. S2. Analysis of *Ab-ps1*, *Ab-tps2* and *Ab-ntre2* transcript relative abundance separately of *A. besseyi* during anoxybiosis or during recovery after keeping anoxybiosis for 1 d.** (A) Transcript level of *Ab-tps1* during anoxybiosis; (B) Transcript level of *Ab-tps2* during anoxybiosis; (C) Transcript level of *Ab-ntre1* during anoxybiosis; (D) Transcript level of *Ab-ntre2* during anoxybiosis; (E) Transcript level of *Ab-atre* during anoxybiosis; (F) Transcript level of *Ab-tps1* during recovery after keeping anoxybiosis for 1 d; (G) Transcript level of *Ab-tps2* during recovery after keeping anoxybiosis for 1 d; (H) Transcript level of *Ab-ntre1* during recovery after keeping anoxybiosis for 1 d; (I) Transcript level of *Ab-ntre2* during recovery after keeping anoxybiosis for 1 d; (J) Transcript level of *Ab-atre* during recovery after keeping anoxybiosis for 1 d. Date is shown as mean with SD (standard deviation). Significant changes are indicated by asterisks (*, $P$<0.05; **, $P$<0.01; $n$=3).

Journal of Experimental Biology • Supplementary information

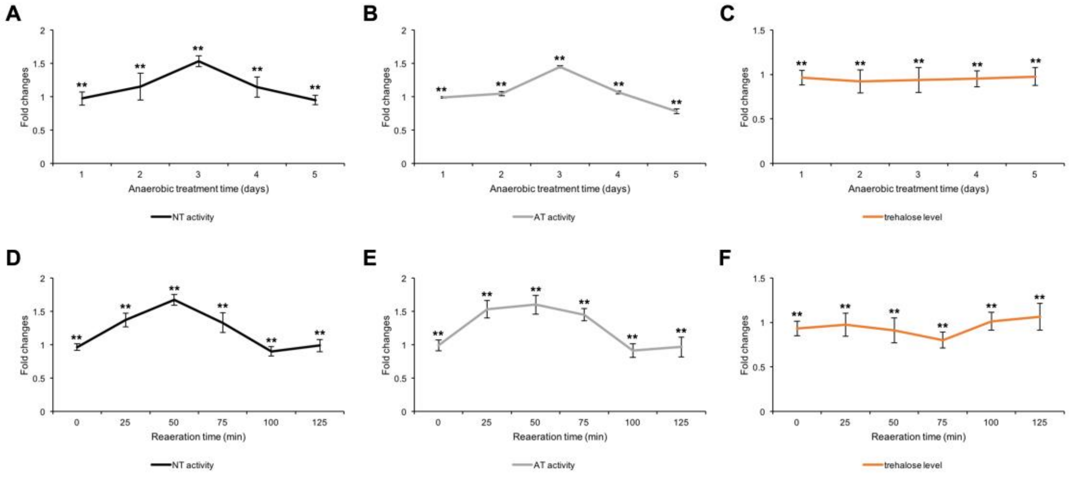


**Fig. S3. Analysis of TRE activity and trehalose level separately of *A. besseyi* during anoxybiosis or during recovery after keeping anoxybiosis for 1 d.** (A) NT activity during anoxybiosis; (B) AT activity during anoxybiosis; (C) Trehalose level during anoxybiosis after keeping anoxybiosis for 1 d; (D) NT activity during recovery after keeping anoxybiosis for 1 d; (E) AT activity during anoxybiosis; (F) Trehalose level during recovery after keeping anoxybiosis for 1 d. Date is shown as mean with SD (**$p$<0.01; *$p$<0.05; $n$=3).

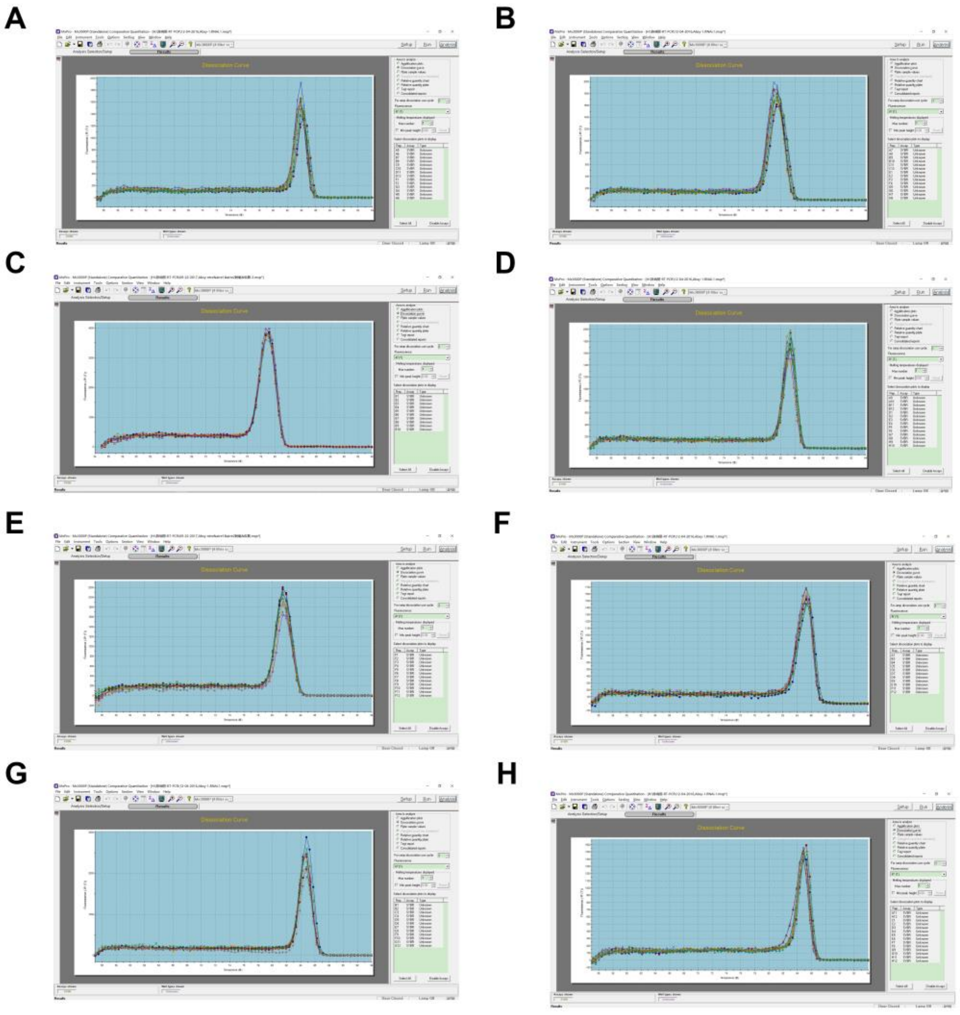


**Fig S4. Melting curves of *Ab-tps1*, *Ab-tps2*, *Ab-ntre1*, *Ab-ntre2*, *Ab-atre*, Ab 28s RNA, *Ab-lea* and *Ab-ace* in RT-qPCR.** (A) Melting curves of *Ab-tps1*; (B) Melting curves of *Ab-tps2*;(C) Melting curves of *Ab-ntre1*; (D) Melting curves of *Ab-ntre2*; (E) Melting curves of *Ab-atre*; (F) Melting curves of Ab 28s RNA; (G) Melting curves of *Ab-lea*; (H) Melting curves of *Ab-ace*.

Journal of Experimental Biology • Supplementary information
